# Transient Electrical and Optical Characteristics of Electron and Proton Irradiated SiGe Detectors

**DOI:** 10.3390/s20236884

**Published:** 2020-12-02

**Authors:** Tomas Ceponis, Laimonas Deveikis, Stanislau Lastovskii, Leonid Makarenko, Jevgenij Pavlov, Kornelijus Pukas, Vytautas Rumbauskas, Eugenijus Gaubas

**Affiliations:** 1 Institute of Photonics and Nanotechnology, Vilnius University, Sauletekio Ave. 3, LT-10257 Vilnius, Lithuania; laimonas.deveikis@tmi.vu.lt (L.D.); jevgenij.pavlov@tmi.vu.lt (J.P.); kornelijus.pukas@tmi.vu.lt (K.P.); vytautas.rumbauskas@ff.vu.lt (V.R.); eugenijus.gaubas@ff.vu.lt (E.G.); 2 Laboratory of Radiation Effects, Scientific-Practical Materials Research Centre of NAS of Belarus, P. Brovki Str. 17, 220072 Minsk, Belarus; lastov@physics.by; 3 Department of Applied Mathematics and Computer Science, Belarusian State University, Independence Ave. 4, 220030 Minsk, Belarus; makleo@mail.ru

**Keywords:** SiGe, radiation detectors, electron and proton irradiations, microwave probed photoconductivity, pulsed barrier capacitance transients, steady-state photo-ionization spectroscopy

## Abstract

The particle detector degradation mainly appears through decrease of carrier recombination lifetime and manifestation of carrier trapping effects related to introduction of carrier capture and emission centers. In this work, the carrier trap spectroscopy in Si_1−x_Ge_x_ structures, containing either 1 or 5% of Ge, has been performed by combining the microwave probed photoconductivity, pulsed barrier capacitance transients and spectra of steady-state photo-ionization. These characteristics were examined in pristine, 5.5 MeV electron and 1.6 MeV proton irradiated Si and SiGe diodes with n^+^p structure.

## 1. Introduction

SiGe material based devices are capable to operate in the radiation harsh environment [1,2,3,4]. It had been demonstrated [5,6] that SiGe alloys are prospective for fabrication of γ-ray detectors. Nevertheless, it has been determined that the Si_1−x_Ge_x_ single crystals can only be obtained for alloys containing either 0.9 < x < 1 or 0 < x < 0.15 of Ge. This alloy usually becomes polycrystalline for the other range of Ge content [6,7]. The radiation damage of the Si_1−x_Ge_x_ devices related with introduction of radiation defects [3,8,9,10,11] can be healed by the atomic reconfiguration of the crystal structure during material anneal [12,13,14]. The particle detector degradation [15,16,17,18] usually appears through decrease of carrier recombination lifetime. Carrier recombination lifetime specifies the fast component of carrier density relaxation within transients of carrier decay via carrier-pair annihilation through deep centers. The decrease of recombination lifetime testifies an enhancement of radiation defect concentration which reduces signals and efficiency of detectors [16,19,20]. The carrier trapping is associated with the single-type carrier emission centers. Capture of the single type carriers to these centers proceeds simultaneously with carrier annihilation, while subsequent thermal emission from these centers delays recombination process. Thereby, the asymptotic component of carrier density relaxation appears in carrier decay transients [20]. The rather shallow levels are usually associated with thermal emission centers which are responsible for the delayed responses of radiation sensors and leakage current [16]. Recombination characteristics of irradiated materials specifies the evolution of radiation defect introduction, their types and densities [19,20]. However, reports on investigations of the carrier recombination characteristics in SiGe alloys irradiated with various species of particles are scarce [12] in literature. In this work, the carrier trap spectroscopy in Si_1−x_Ge_x_ structures, containing 1 and 5% of Ge, have been performed by combining several contact and contactless techniques. The microwave probed photoconductivity, pulsed barrier capacitance transients and spectra of steady-state photo-ionization [20] in pristine, 5.5 MeV electron and 1.6 MeV proton irradiated Si and SiGe diodes with n^+^p structure were examined. It has been revealed that both carrier recombination and trapping lifetimes decrease near-reciprocally to density of radiation defects acting as carrier capture and thermal emission centers, with predominance of point radiation defects. It has been hypothesized that the single-type deep centers are involved in both carrier photo-generation and thermal emission processes. Activity of point defects can be modified by anneal procedures, and sensor functionality can be recovered (at least, partially). On the other hand, Ge can be used as an isovalent impurity, instead of carbon, within engineering of the strong field layers in advanced low gain avalanche detectors made of Si.

## 2. Samples and Experimental Techniques

The radiation detectors under tests were fabricated at Scientific-Practical Materials Research Centre of NAS of Belarus in a configuration of n^+^p diodes using SiGe substrates grown by Czochralski technique. The diodes contained the 2 × 3 mm^2^ surface area and 500 μm thickness. Several Si_1−x_Ge_x_ single-crystal alloys with bandgap *E_G_* varied according to x as [21] *E_G_* = 1.12 − 0.41x + 0.008x^2^ (eV) for x < 0.85 (at 300 K) were fabricated by incorporating either 1 or 5% of Ge. The diode basis was formed of p-type Si_1−x_Ge_x_ material which conductivity was controlled by concentration of boron dopants. The p-Si based diodes with the same doping were examined for comparison. Irradiations with 5.5 MeV electrons at room temperature were implemented by using a linear electron accelerator at Scientific-Practical Materials Research Centre of NAS of Belarus. Different exposures at fixed electron flux of 2 × 10^12^ cm^−2^s^−1^ were applied to vary electron irradiation fluence. Irradiations with 1.6 MeV proton beam were performed by a Tandetron 4110A accelerator at Center for Physical Sciences and Technology, Lithuania, gradually increasing fluence. Proton beam current was varied in the range of 20–40 nA, and the largest fluence up to 10^15^ p/cm^2^ was collected under the longest exposure. The pristine (A_p_, B_p_ and C_p_), electron (A_e_, B_e_ and C_e_) and proton (A_P1_, B_P1_ and C_P1_) irradiated Si and SiGe diodes, respectively listed in Table 1, were examined.

The routine capacitance-voltage (C-V) characteristics were examined to control dopant concentration. These characteristics were measured at dc bias voltage (≤5 V) using a 1 MHz frequency harmonic test signal, before and after irradiation with definite fluence. The C-V characteristics were recorded employing a QuadTech 7600B LRC-meter.

The pulsed capacitance technique has been applied for junction barrier evaluation by linearly increasing voltage (BELIV) [20,22,23], and for estimation of carrier emission lifetime in the pristine and irradiated diodes. The BELIV circuitry contains a pulse generator (any generator of special waveforms) of the linearly increasing voltage *U_p_*(*t*) *= At* which is connected in series with a diode under test and a load resistor *R_L_*. The barrier capacitance (*C_b_*) charging current transients *i_C_*(*t*) are recorded by an Agilent DSO6102A oscilloscope (purchased from Agilent Company, Santa Clara, CA, USA) using the 50 Ω load input. Routinely, the charging current transients of the reverse biased diode are recorded in dark. To vary the filling of the carrier emission centers, the BELIV transients can be measured under bias illumination using either steady-state or short laser pulses. The linearly increasing voltage *U*(*t*) modifies the barrier capacitance *C_b_*(*t*) = *C_b0_*(1 + *U*(*t*)*/U_bi_*)^−1/2^. Charging of the barrier capacitance *C_b_*(*t*), which varies in time (*t*), induces a current *i_C_*(*t*). Here, *C_b_*_0_ *= εε*_0_*S/w*_0_ *=* (*εε*_0_*S*^2^*eN_A_*/2*U_bi_*)^1/2^ is the barrier capacitance of the non-biased diode of an area *S*, *ε*_0_ is a vacuum permittivity*, ε* is material dielectric permittivity, *e* is elementary charge, *U_bi_* is the built-in potential barrier, *w*_0_ = (2*εε*_0_*U_bi_*/*eN_A_*)^1/2^ is a width of depletion due to the built-in barrier and dependent on dopant concentration *N_A_*, *A = U_P_*/*τ_PL_* is the ramp of linearly increasing voltage (LIV) pulse with a *U_P_* peak amplitude and *τ_PL_* duration. The current transient *i_C_*(*t*) in the external circuit, determined by the time dependent barrier capacitance changes, is represented as [22]:

(1) $$i_{C}\left(t\right)=\frac{dq}{dt}=\frac{\partial U}{\partial t}\left(C_{b}+U\frac{\partial C_{b}}{\partial U}\right)=\frac{\partial U_{C}}{\partial t}C_{b0}\frac{1+\frac{U_{C}\left(t\right)}{2U_{bi}}}{{\left(1+\frac{U_{C}\left(t\right)}{U_{bi}}\right)}^{3/2}}\approx AC_{b0}\frac{1+\frac{At}{2U_{bi}}}{{\left(1+\frac{At}{U_{bi}}\right)}^{3/2}}$$

Then, the current transient *i_C_*(*t*) contains an initial (*t* = 0) step followed by a descending component. The initial current step *i_C_*(0) = *AC_b_*_0_ is associated with the displacement current, and it is usually delayed by *R_L_C_b_*. Therefore, the *i_C_*(*t*) can be estimated by using convolution *i_C_*(*t*) *=* (*R_L_C_b_*_0_)^−1^*∫*_0_*^t^ i_C_*(*x*)exp[−(*t−x*)/(*R_L_C_b_*_0_)]*dx* integral. The vertex amplitude of the initial current step is proportional to a value of a ramp *A* of LIV pulse. The ramp *A* can be controlled by differentiating LIV pulse. The peak value of the *i_C_*(*t*) is a reciprocal of the depletion width, which is modulated by the excess carrier concentration at the moment of appearance of a LIV pulse. The descending component of BELIV transients is usually governed by the charge extraction. Thereby, the changes of the shape of the diode-inherent BELIV transients are determined by instantaneous densities of carriers, and the components of transients indicate manifestation of carrier recombination and thermal emission centers. In the structures containing high concentration of emission centers, the carriers thermally emitted from these traps result in the formation of the charge-collection phase of a BELIV pulse. The corresponding generation current is

(2) $$i_{g}\left(t\right)=en_{i}Sw_{0}{\left(1+U\left(t\right)/U_{bi}\right)}^{1/2}/\tau_{g}$$

(here, *n_i_* is the intrinsic density of carriers and *τ_g_* is the carrier lifetime relative to thermal emission), and it determines the barrier recharging due to carriers emitted into free carrier band and appears within a rearward phase of the transient as the increasing component of the BELIV transient under either illumination or resonant LIV pulse duration (*τ_PL_*), relative to *τ_g_*. The *i_g_*(*t*) is accounted for by an increase of the volume from which the carriers are collected by the increased depletion width during the reverse-polarity LIV pulse. This current increases with *U*(*t*) within LIV pulse and can exceed the barrier charging current in the rearward phase of the transient. The transient of the total reverse current is a sum of the currents

(3) $$i_{R}\left(t\right)=i_{C}\left(t\right)+i_{g}\left(t\right)$$

The steady-state photo-ionization spectroscopy (SS-PIS) [20,23] was applied to highlight the photo-active centers in the pristine and irradiated diodes with various content of Ge within SiGe alloy. The photometric 800 W lamp served as a source of varied wavelength which light was dispersed through a double-path monochromator DMR4. Thereby, the excitation wavelength was varied in the range of 0.5–3 μm. The Keithley 2635B source-meter was employed to measure the dc photo-current induced by varied excitation wavelength. The sample under test was mounted in a cryostat and cooled by liquid nitrogen to decrease the impact of phonons and leakage current. A photo-ionization spectrum usually shows a step-like structure. The photo-ionization step can be approximated by the Kopylov-Pikhtin model [24,25]:

(4) $$\sigma_{e-ph}\propto \int_{0}^{\infty }\frac{e^{-\frac{{\left(E+E_{dl}-h\nu \right)}^{2}}{\Gamma^{2}}}}{h\nu {\left(E+E_{dl}\right)}^{2}}\sqrt{E}dE$$

Here, *σ_e-ph_* is the photo-ionization cross-section, as a parameter integrated over spectrum of electron energies *E*, *E_dl_* is the photo-activation energy of a definite center and the electron-phonon coupling is defined by the broadening factor *Γ*. The photo-generated carriers of density *N*(*hυ*) induces a photo-current *i*(*hυ*) dependent on excitation photon energy *hυ*:

(5) $$i\left(h\nu \right)=\frac{q_{e}N\left(h\nu \right)S\mu U_{R}}{d}$$

where *q_e_* is the elementary charge, *S* is the junction area, *d* is the diode base thickness, *μ* is the carrier mobility and *U_R_* is the reverse bias voltage.

To estimate density of carrier recombination and thermal emission centers, introduced by electron and proton irradiations, the carrier characteristic lifetimes have been extracted from the recorded carrier decay transients, as a slope of carrier density relaxation curves. These evaluations were performed by employing the microwave-probed photoconductivity (MW-PC) transients recorded ex-situ and analyzed as a function of irradiation fluence. The carriers were excited by infrared 1062 nm laser with 400 ps pulses, and excited area was probed by 22 GHz microwaves using a slit antenna. The transients were recorded by Tektronix TDS-5104 oscilloscope (purchased from Tektronix company, Bracknell, Berkshire, UK) using 50 Ω load resistor. The single exponential MW-PC decays were usually observed in the as-irradiated diodes. This indicates that the linear recombination prevails (with a single slope of the transient displayed within a semi-log scale). The deep traps act as the recombination defects, while carriers are temporary captured on shallow levels which act as the trapping (generation current) centers. The carrier recombination lifetime (*τ_R_*) was measured within the initial MW-PC decay stage at e^−1^ level relative to a peak value of the transient. A few transients showed a two-componential decay containing the asymptotic decay component, which is related to the trapping lifetime (*τ_tr_*). The two-componential decay is there caused by the multi-trapping effect. This two-componential transient appears when carrier lifetime depends on the excess carrier density. The thermally generated carriers from the emission centers, vary the concentration of the recombining carriers and delay the carrier-pair annihilation process. The latter carrier-pair annihilation process prevails in the initial stage of carrier decay. The initial decay is ascribed [20] to recombination lifetime *τ_R_* while asymptotic decay is characterized by the instantaneous trapping lifetime *τ_inst,tr_* related to trapping coefficient *K_tr_* as

(6) $$\tau_{inst,tr}=\tau_{R}K_{tr};\;\;K_{tr}=1+\frac{T_{tr}N_{C,V,e,h,Ttr}}{{\left(N_{C,V,e,h,Ttr}+\Delta n\right)}^{2}}$$

where *T_tr_* is a concentration of shallow trapping centers, *N_C_*_,*V*,*e*,*h*,*Ttr*_ = *N_C_*_,*V*_exp(*−*Δ*E_Ttr_*/*kT*) is the effective density of band states, and Δ*n* is the trapping attributed excess carrier density. The quasi-exponential decay with time (*t*) dependent instantaneous lifetime *τ_inst_*_,*tr*_(Δ*n*(*t*)) is a reason of the non-linear carrier decay process.

The sample depth dependent carrier decay lifetime appears when sample thickness is larger than radiation stopping range. Such a situation appeared under diode irradiation with 1.6 MeV protons. To overcome profiling of carrier lifetime, the near-surface excitation regime has been applied, and carrier decay transients were examined in-situ during proton irradiations. Therefore, the sample with the probes were mounted within vacuum irradiation chamber. Then carriers were excited by strongly absorbed 532 nm laser light using 400 ps pulses. The sample-edge excited area [20] of effective depth *d_eff_*, related to an absorption coefficient (α_532_^−1^ ~ *d_eff_*,) of the diode material at the excitation wavelength, was probed by 22 GHz microwaves using a needle-tip coaxial antenna. The transients were recorded by DSO6102A oscilloscope using 50 Ω load resistor. The two-componential decay can also appear in such a situation. However, the short decay component should be attributed to transitional processes due to surface recombination, while asymptotic relaxation is ascribed to the effective carrier decay lifetime composed of the surface *τ_s_* and the bulk *τ_R_* recombination, as [20]:

(7) $$\tau_{eff}^{-1}=\tau_{R}^{-1}+\tau_{s}^{-1}$$

The *τ_eff_* ≈ *τ_R_* when *τ_s_ >> τ_R_*. To implement such a regime, the excitation fiber and MW needle-tip antenna are used. These probes are then located at the predicted (through TRIM simulations) stopping depth relatively to the sample surfaces.

## 3. Parameters Extracted from Transient Electrical and Optical Characteristics

The dopant concentrations (*N_S_*) in the diode base region were evaluated using the measured C–V characteristics. These parameters in the pristine and irradiated samples are listed in Table 2. The experimental errors in evaluation of dopant concentration *N_S_* do not exceed 2% of the extracted values. It can be deduced from Table 2 that the effective dopant concentration in Si and Si_0.99_Ge_0.01_ material diodes decreases with enhancement of irradiation fluence. This can be explained by introduction of donor type radiation defects which compensate boron dopants.

However, the opposite tendency of doping level variation with irradiation fluence was revealed for the Si_0.95_Ge_0.05_ material diodes. This might be explained either through prevailing of the acceptor-like radiation defects in p-type Si_0.95_Ge_0.05_ material or via lattice bond length variations which affect the conduction and valence band parameters of the SiGe alloy [26] and the consequent shifts of Fermi level. The thermal activation energy shifts of radiation induced deep traps to low values with increase of Ge content in the SiGe alloy were inferred using DLTS characteristics obtained on the same diodes and published elsewhere. This result supports an assumption of lattice bond length variations with Ge content [27].

**Figure 1:**
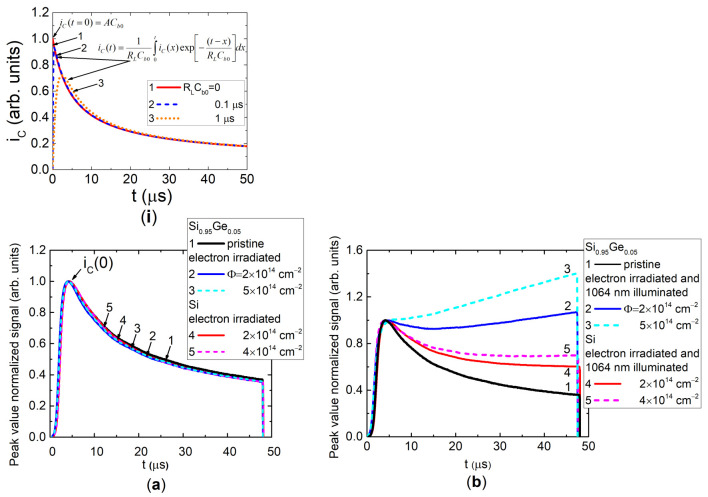
Evolution of BELIV transients in Si as well as Si_0.95_Ge_0.05_ diodes irradiated with 5.5 MeV electrons using various fluences and recorded in dark (**a**) as well as under 1064 nm wavelength laser illumination. (**b**) Here, LIV pulses of *U_P_* = 12 V and *τ_PL_* = 48 μs were employed. In the inset (**i**), the BELIV pulse components are denoted. Please mark the corresponding serial number in the figure.

The transients of the barrier capacitance pulsed charging (BELIV) might be useful in estimation of the thermal emission centers introduced by doping and irradiation procedures. The BELIV transients recorded on Si as well as Si_0.95_Ge_0.05_ diodes irradiated with 5.5 MeV electrons and rather moderate fluences are illustrated in Figure 1a. These transients are peak-current normalized to exclude an impact of diode area and absolute value of barrier height. The rather short LIV pulses (of *τ_PL_* = 48 μs) are there employed to have considerably large charging currents (due to elevated ramp *A = U_P_*/*τ_PL_* of LIV pulses of the invariable *U_P_* = 12 V amplitude). The shape of transients in Figure 1a manifests only the impact of barrier capacitance charging where an initial step *AC_b0_* represents the displacement current (*i_C_*(*t*)), delayed due to measurement circuit *R_L_C_b0_*, and a descending component governed by the subsequent charge extraction current. Therefore, the *i_C_*(*t*) can be estimated by using a convolution *i_C_*(*t*) = (*R_L_C_b_*_0_)^−1^*∫*_0_*^t^ i_C_*(*x*)exp[−(*t − x*)/(*R_L_C_b0_*)]*dx* integral. The impact of the thermal emission centers can be highlighted by additional illumination (Figure 1b) implemented using IR (1064 nm) laser. This additional illumination changes filling of the thermal emission centers. The laser generated excess carriers of density *n_ex_* may change the effective density of band states as *N_C,eff_* ≅ *N_C_ − n_ex_*, and, thereby, thermal emission time *τ_g_* = exp(*E_tr_*/*kT*)/*v_th_σN_C_*_,*eff*_ (in Equation (2)). Here, *E_tr_* is an activation energy of trap, *kT* is the thermal energy at temperature *T*, *v_th_* is thermal velocity of trapped carriers, *σ* is the carrier capture/emission cross-section. Thus, reduction of *N_C_*_,*eff*_ leads to an increase of *τ_g_*, which approaches to *τ_PL_* and highlights the trapping/thermal emission centers. Additionally, the deeper an emission center (relative to the activation energy *E_tr_*) the longer *τ_g_* is.

Figure 1b illustrates how the irradiation and additional illumination changes the BELIV transients of Si and Si_0.95_Ge_0.05_ diodes. The radiation introduced emission centers increase the generation lifetime *τ_g_*~1/*N_C,eff_*, due to reduction of the *N_C_*_,*eff*_ by the released carriers, as *N_C_*_,*eff*_ ≅ *N_C_* − *n_ex_.* Also, the generation current increases, as the enhanced density *m_tr_* of emission centers elevates the i_g_^*^~*em_tr_Sw*/*τ_PL_*, when the i_g_^*^ value exceeds that of *i_C_* (see Equation (3)). Thereby, the enhancement of the electron irradiation fluence determines the increase of the thermal emission current in both Si and Si_0.95_Ge_0.05_ diodes (Figure 1b). However, density *m_tr_* of the radiation induced emission centers seems to be dependent on the single crystal matrix. The proportionality of the i_g_^*^ to *m_tr_* is masked by the competition of i_g_^*^ and *i_C_* components within *i_R_* (Equation (3)), and the observed values of *i_g_* significantly depend of the range of *τ_PL_*. Thereby, the qualitative estimation of thermal emission centers can reliably be elucidated using BELIV transients. Nevertheless, assuming *τ_g_→τ_PL_* for transients shown in Figure 1b, the *m_tr_* introduced by electron beam into Si_0.95_Ge_0.05_ diodes is 1.8 times larger relative to that of Si material diode, at the same collected fluence of 2 × 10^14^ e/cm^2^. On the other hand, it can testify that the prevailing thermal emission centers in Si_0.95_Ge_0.05_ diodes are shallower by 15 meV than those, radiation induced into Si diodes. The latter result is in line with DLTS results (obtained for the same Si and Si_0.95_Ge_0.05_ diodes) due to thermal activation energy shifts of radiation induced deep traps to low values with enhancement of Ge content within SiGe alloy.

**Figure 2:**
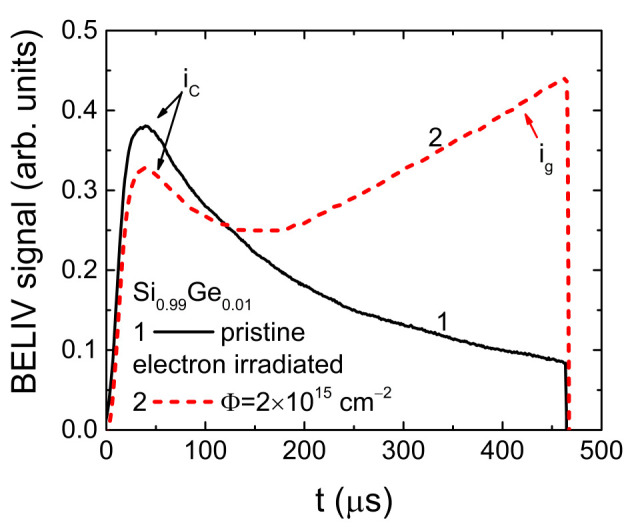
The BELIV transients recorded in pristine and electron irradiated Si_0.99_Ge_0.01_ diodes using LIV pulses of *U_P_* = 12 V and *τ_PL_* = 470 μs.

The changes of carrier thermal generation lifetime can be directly observed in heavily irradiated diodes with significantly increased *m_tr_* values when *τ_g_→τ_PL_* through elongated *τ_PL_* and i_g_^*^→i_C_ due to reduction of the ramp *A* of long LIV pulses. Such a situation had been materialized in Si_0.99_Ge_0.01_ diodes irradiated with 2 × 10^15^ e/cm^2^ 5.5 MeV electrons, as illustrated in Figure 2. There, the BELIV transients in the pristine and irradiated diodes are compared. It can be clearly noticed, that the initial peak value in the BELIV transient is reduced for the irradiated diode due to the excess carrier capture. There, the decrease of ramp *A* for long LIV pulses should be coordinated with the τ*_PL_* range to get *i_g_* ≥ *i_C_* and to directly highlight the *i_g_* component. Direct separation of the *i_g_* component enables estimation of the *τ_g_* and *E_tr_* values. Assuming the inherent values of *v_th_* = 10^7^ cm/s, *σ* = 10^−14^ cm^2^, and *N_C_* = 10^19^ cm^−3^ in Si and SiGe at room temperature, the *E_tr_* = 0.47 eV was extracted using the transients of Figure 2. This would imply the traps with deep levels in the mid-bandgap.

**Figure 3:**
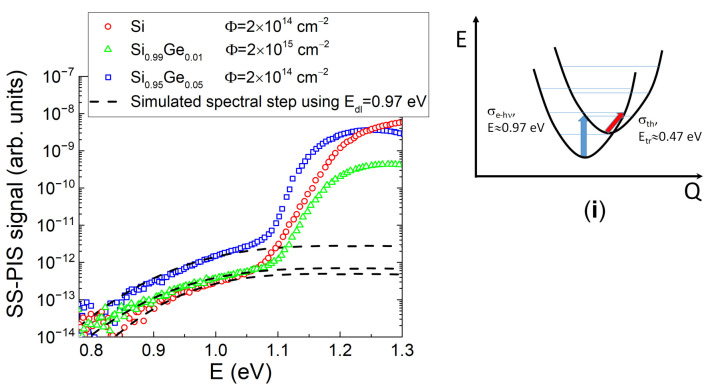
SS-PIS spectra recorded in the electron irradiated Si and SiGe diodes. In the inset (**i**), the configuration diagram sketched using SS-PI spectra and BELIV estimations, made using Figure 1 and Figure 2.

The recorded SS-PI spectra (Figure 3) really represent the step-like structure containing a single step, where spectral variation electron-photon interaction cross-section is well approximated by the Kopylov-Pikhtin model, represented by Equation (4). The spectral step peaked in the range of 1.1–1.3 eV, being above bandgap of *E_G_* = 1.1 eV for the SiGe alloys, might appear due to the bandgap absorption modified by surface recombination. This assumption is supported by the changes of SS-PIS signal reduction in the range of >1.2 eV for both SiGe alloys. This SS-PIS signal decrease develops noticeably with Ge content (Figure 3). The simulated spectral steps, made using this Equation (4) and assuming values of the broadening factor in the range of *Γ* = 0.119–0.120, and shown in Figure 3 by dashed-curves. Superposition of the measured and simulated spectral steps implies that a single deep photo-active center with activation energy of *E_dl_* = 0.97 eV prevails in all the electron irradiated samples irrespective of Ge content. Nevertheless, values of the photo-current (height of spectral steps) appear to be slightly dependent on irradiation fluence and Ge content within SiGe alloy. This effect can be explained by fluence dependent excess carrier concentration, generated by electron-photon interaction within SS-PIS process. The SS-PIS photo-current should be proportional to this carrier concentration (according to Equation (5)), which seems to be modulated by carrier capture and recombination processes. Thereby, a SS-PIS step obtained in SiGe diodes irradiated with 2 × 10^14^ e/cm^2^ fluence appears to be the higher one relative to that irradiated with 2 × 10^15^ e/cm^2^ fluence (Figure 3). Differences of relative heights of the SS-PIS steps obtained in the electron irradiated Si and SiGe alloys can be explained by activation energy shifts of radiation induced deep traps with enhancement of Ge content within SiGe alloy. Assuming a simplified approach, where these single-type deep centers are involved in both carrier photo-activation (SS-PIS, Figure 3) and thermal emission (within BELIV transients, Figure 1b and Figure 2), a configuration diagram might be sketched (see the inset *i* for Figure 3).

**Figure 4:**
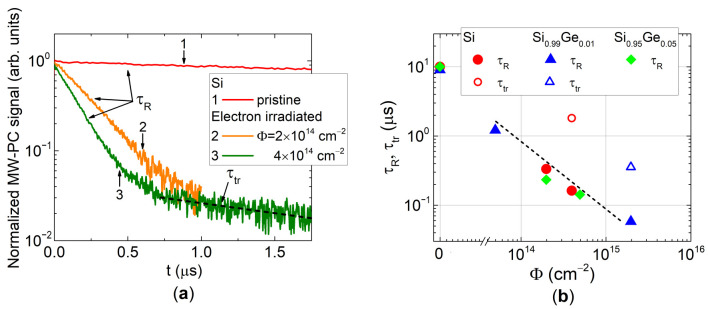
(**a**) Carrier decay transients recorded by MW-PC technique in Si diodes irradiated with 5.5 MeV electrons collecting different fluence. (**b**) Carrier lifetimes ascribed to their decay through recombination and trapping centers as a function of electron irradiation fluence in pristine and irradiated Si and SiGe samples (A, B and C, Table 1).

Modulation of the concentration of the photo-excited excess carriers by carrier capture and recombination processes was examined using analysis of the photo-conductivity transients. Carrier lifetime in pristine and electron irradiated samples was extracted after irradiations using microwave-probed photoconductivity (MW-PC) transients (Figure 4a). The deep traps act as the recombination defects. However, the shallow levels act as the trapping (generation current) centers [20], which temporary capture the excess carriers followed by further thermal trapped carrier release into free states. The carrier recombination lifetime (*τ_R_*) was measured within the initial MW-PC decay stage at e^−1^ level relative to a peak value of the transient (Figure 4a). Some transients (in samples irradiated with large fluence) contained the second (asymptotic decay) component, which is related to the trapping lifetime (*τ_tr_*), Equation (6). Values of both carrier lifetimes (*τ_R_* and *τ_tr_*) as a function of electron irradiation fluence are represented in Figure 4b.

It can be noticed in Figure 4b that trapping lifetimes (*τ_tr_*) are nearly an order of magnitude longer than the recombination lifetimes. Also, the trapping effect can be resolved reliably only in heavily irradiated diodes when radiation defect concentrations are sufficiently large. Both carrier recombination and trapping lifetimes decrease near-reciprocally relative to density of the radiation defects acting as carrier capture and thermal emission centers. Variation of values of the carrier recombination lifetimes in the electron irradiated Si and SiGe diodes obeys the same line (eye-guided by dot curve in Figure 4b) in a double-log *τ_R_* − *Φ* scale. This hints on nearly the same mechanisms of radiation damage of the examined Si and SiGe materials.

**Figure 5:**
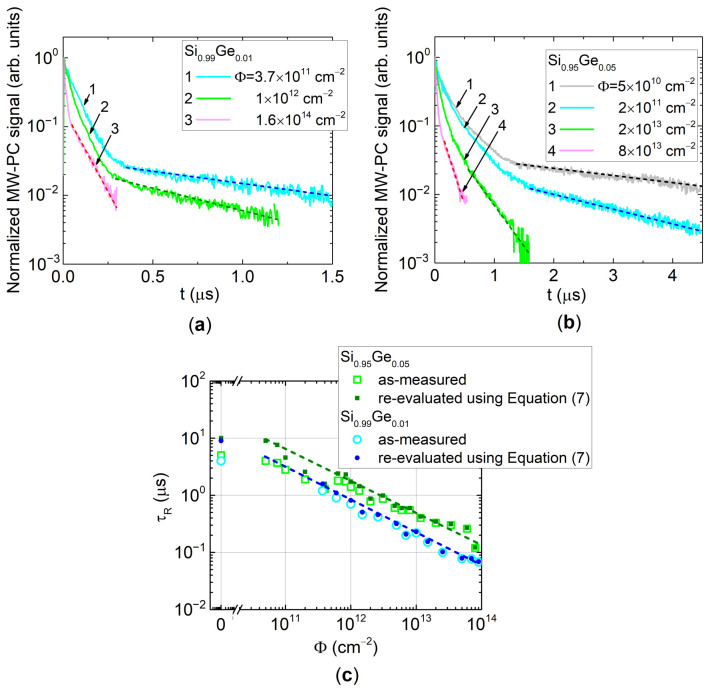
The MW-PC relaxation transients recorded on Si_0.99_Ge_0.01_ (**a**) and Si_0.95_Ge_0.05_ (**b**) diodes irradiated with 1.6 MeV protons of varied fluence. (**c**) Carrier recombination lifetime as a function of 1.6 MeV proton fluence in Si_0.99_Ge_0.01_ and Si_0.95_Ge_0.05_ samples. Here, the MW-PC transients shown in (**a**,**b**) were in situ scanned during proton irradiation within stopping range of 1.6 MeV protons.

The more complicated situation (relatively to that of penetrative electrons, illustrated in Figure 4) appears in recording and analysis of the MW-PC transients after and during irradiation with stopped particles, namely 1.6 MeV protons. There, the sample depth dependent carrier decay lifetime is inherent. As mentioned, the near-surface excitation regime has been applied, and carrier decay transients recorded within stopping range of 1.6 MeV protons were examined in-situ during proton irradiations, to overcome profiling of carrier lifetime. The recorded transients appear also (like in the case of carrier trapping) to be similar to the two-componential decay (Figure 5a,b). However, the initial decay fragment of the transient should be ascribed to the transitional processes of surface recombination [20], due to inhomogeneous excitation by strongly absorbed light. The asymptotic decay is then governed by the surface (*τ_s_*) and bulk recombination (*τ_R_*) lifetimes. The latter parameter is really modified by radiation defects, and transients (illustrated in Figure 5a,b) significantly vary depending on the collected proton irradiation fluence. The surface (*τ_s_*) recombination lifetime has been additionally estimated by dedicated investigations using a technique of several excitation wavelengths [20]. Then, values of the carrier bulk recombination lifetime (Figure 5c), attributed to proton introduced radiation defects, were corrected using these *τ_s_* and the as–measured *τ_R_* − *Φ* characteristics, by applying Equation (7). It can be inferred from Figure 5c that corrections of the *τ_R_* values are important only in the range of small fluences where *τ_s_* is close to those of *τ_R_* values. The corrected *τ_R_* − *Φ* characteristics, obtained for diodes made of SiGe alloy with different Ge content, appear to be the near-reciprocally dependent on density of the radiation defects, assumed being proportional to the proton irradiation fluence *Φ*. As can be deduced from Figure 5c, the carrier recombination lifetime, governed by radiation defects, is obtained to be longer for the SiGe alloy with the larger Ge content (of 5%) than that of 1% Ge. This result implies that the SiGe alloys (with enhanced Ge content) can be prospective materials in fabrication of radiation tolerant particle (especially hadron) detectors. However, a trade-off for Ge content enhancement within SiGe material and growth of the single-crystal SiGe alloys should be found.

## 4. Summary

The radiation hardness of SiGe alloys relative to penetrative (5.5 MeV electrons) and strongly absorbed (1.6 MeV protons) high energy particles has been estimated by control of carrier emission and capture centers characterized by carrier recombination as well as thermal emission lifetime variations dependent on irradiation fluence. These characteristics have been recorded in diodes made of SiGe alloys with different Ge content and compared with those obtained for Si of the same p-type conductivity and doping level.

It has been demonstrated that qualitative estimation of thermal emission centers can be implemented using BELIV transients related to barrier capacitance of the diodes. Nevertheless, the density of trapping centers introduced by electron beam into Si_0.95_Ge_0.05_ diodes might be 1.8 times larger relative to the Si material diode, at the same collected fluence. On the other hand, it can testify that the prevailing thermal emission centers in Si_0.95_Ge_0.05_ diodes are shallower by 15 meV than those, radiation induced into Si diodes. The latter result is in line with DLTS results obtained for the same Si and Si_0.95_Ge_0.05_ diodes due to thermal activation energy shifts of radiation induced deep traps to low values with enhancement of Ge content within SiGe alloy. It has been demonstrated that separation of the generation current components within BELIV transients (when possible) enables estimation of the thermal emission lifetime and, consequently, thermal activation energy *E_tr_*. The activation energy of *E_tr_* = 0.47 eV was extracted from BELIV transients for the prevailing species of the radiation defects. The close values of activation energy in the range of 0.5–0.58 eV had been revealed by the dedicated investigations of DLTS spectra in the irradiated Si and SiGe diodes [11,13]. The radiation defects with such the activation energy were assigned to the radiation induced complexes of interstitials with carbon or boron impurities. Thereby, the revealed prevailing emission centers in our work are in line with DLTS data published in literature. The prevailing photo-generation centers with photo-activation energy of 0.97 eV have been also revealed using the steady-state photo-ionization spectroscopy. Assuming a simplified approach, where the single-type deep centers are involved in both carrier photo-ionization and thermal emission, a configuration diagram for the carrier capture-release processes has been sketched. It has been revealed that both carrier recombination and trapping lifetimes decrease near-reciprocally relative to density of radiation defects acting as carrier capture and thermal emission centers. It has been found that variation of the recombination lifetimes in the irradiated Si and SiGe diodes obeys the same type curve in the double-log *τ_R_* − *Φ* scale. This hints on nearly the same mechanisms of radiation damage of the examined Si and SiGe materials. A technique of measurements, implemented by locating the fiber and needle-tip MW probes within 1.6 MeV proton stopping range, has been applied to examine fluence dependent lifetime characteristics under irradiations by strongly absorbed particles. It has been revealed that the carrier recombination lifetime, governed by radiation defects introduced using the strongly absorbed proton irradiation, is longer for the SiGe alloy containing the larger Ge content (of 5%). This might be explained through prevailing of the acceptor-like radiation defects in p-type SiGe alloy material containing the enhanced content of Ge. Also, it has been shown the predominance of point radiation defects. Activity of point defects can be modified by anneal procedures and, thereby, recovery of irradiated sensors can be performed. The SiGe alloys might also be promising in engineering of the strong field layer of the advanced low gain avalanche detectors [28].

## Figures and Tables

**Table 1:** The diodes under test, made on p-type Si and SiGe material substrates of the same doping.

**Diode Base Material**	Si			Si_0.99_Ge_0.01_			Si_0.95_Ge_0.05_		
**Diode Batch**	A			B			C		
**Pristine Material**	(A_p_) Pristine			(B_p_) Pristine			(C_p_) Pristine		
**5.5 MeV Electron Irradiation Fluences**		(A_e1_) 2 × 10^14^ e/cm^2^	(A_e2_) 4 × 10^14^ e/cm^2^		(B_e1_) 5 × 10^13^ e/cm^2^	(B_e2_) 2 × 10^15^ e/cm^2^		(C_e1_) 2 × 10^14^ e/cm^2^	(C_e2_) 5 × 10^14^ e/cm^2^
**1.6 MeV Proton Irradiation Fluences**		(A_P1_) 0–10^15^ p/cm^2^			(B_P1_) 0–10^15^ p/cm^2^			(C_P1_) 0–10^15^ p/cm^2^	

**Table 2:** The doping concentrations evaluated using C-V characteristics.

Sample		*N_S_* (cm^−3^)	*N_S_* (cm^−3^) in Proton Irradiation Experiments
Diode Base Material	Fluence *Φ* (e/cm^2^)		
Si	Pristine (*Φ* = 0)	1.93 × 10^14^	
	2 × 10^14^	1.76 × 10^14^	
	4 × 10^14^	1.69 × 10^14^	
Si_0.99_Ge_0.01_	pristine	1.82 × 10^15^	6.61 × 10^13^
	5 × 10^13^	n/a	
	2 × 10^15^	1.45 × 10^15^	
Si_0.95_Ge_0.05_	pristine	1.81 × 10^14^	2 × 10^14^
	2 × 10^14^	1.86 × 10^14^	
	5 × 10^14^	1.89 × 10^14^	
